# Multimodal hard X-ray nanoprobe techniques for *operando* investigations of photovoltaic devices

**DOI:** 10.1107/S1600577525006034

**Published:** 2025-08-19

**Authors:** Eunyoung Choi, Sarah Wieghold, Carlo A. R. Perini, Yanqi Luo, Sanggyun Kim, Juan-Pablo Correa-Baena, Samuel D. Stranks, Julia E. Parker

**Affiliations:** a Diamond Light Source Harwell Science and Innavation Campus DidcotOX11 0DE United Kingdom; b Department of Chemical Engineering and Biotechnology University of Cambridge Philippa Fawcett Drive CambridgeCB3 0AS United Kingdom; c Advanced Photon Source Argonne National Laboratory Lemont IL60439 USA; d School of Materials Science and Engineering Georgia Institute of Technology Atlanta GA30332 USA; University College London, United Kingdom

**Keywords:** hard X-ray nanoprobes, XBIC, XRF, perovskite solar cells

## Abstract

Compared with conventional laboratory-scale X-ray techniques, synchrotron based X-rays with higher brilliance and higher coherence allow for the investigation of various material properties with high spatial resolution. The microscopic behaviours of materials can be examined using the Hard X-ray Nanoprobe beamline (I14) at Diamond Light Source, which provides a 50 nm focused beam and has been successfully employed to identify nanoscale optoelectronic features in energy-harvesting materials such as halide perovskites that exhibit local heterogeneity. We have developed X-ray beam-induced current (XBIC) measurement capability at I14 to address the growing demand for *operando* analysis in energy-harvesting research. Here, we demonstrate that X-ray fluorescence (XRF)/XBIC multimodal measurements are feasible at I14 and apply these newly implemented techniques to study perovskite solar cells with various additive concentrations to understand the effect of the additive on nanoscale optoelectronic performance. This expanded *operando* characterization capability offers the possibility of monitoring nanometre-scale compositional variations and corresponding optoelectronic features of actual solar cell configurations.

## Abstract 2

X-ray beam-induced current (XBIC) measurements were implemented on the Hard X-ray Nanoprobe at Diamond Light Source, allowing simultaneous XBIC and nano X-ray fluorescence mapping of photovoltaic devices.

## 1. Introduction

To mitigate the adverse environmental impact of fossil fuels while meeting global electricity demands, it is essential to replace them with clean energy sources that offer high-power conversion efficiency. Among various alternatives, photovoltaic cells are a promising alternative, due to the abundance of solar energy. However, to obtain highly efficient devices with stability from such abundant resources, a prerequisite is an understanding of the materials used in photovoltaic solar cells. Synchrotron based characterization using nano-focused coherent X-rays with high photon intensity allows for the investigation of concealed features in energy-harvesting materials (Szostak *et al.*, 2022 ▸; Zhou *et al.*, 2020 ▸).

At the Hard X-ray nanoprobe beamline (I14) at Diamond Light Source (DLS), the X-ray beam can be focused to 50 nm, enabling various scanning X-ray techniques such as X-ray fluorescence (XRF), X-ray diffraction (XRD) and X-ray absorption spectroscopy with high spatial resolution (Quinn *et al.*, 2021 ▸). Using XRF, the influence of compositional heterogeneity in solution-processed (Frohna *et al.*, 2022 ▸) or evaporated perovskites (Chiang *et al.*, 2023 ▸) was discovered, while XRD revealed distinct diffractions attributed to tetragonal superstructure in microscale regions of interest (Doherty *et al.*, 2021 ▸). These studies demonstrate the importance of analysis techniques using nano-focused scanning X-ray microscopy, particularly for perovskite based photovoltaic devices where local chemical and structural heterogeneity is rife.

The continuous development of improved methodologies with the nanoprobe beamline is essential for performing *operando* characterization for optoelectronic devices, which enables observation of nanoscale phenomena within device configurations (Szostak *et al.*, 2023 ▸). In response to a growing demand for such technology advancements, various beamlines with a nano-focused beam have expanded their capability to allow *operando* analysis for energy-harvesting devices with multimodal X-ray techniques. For instance, the effect of Cu diffusion in cadmium telluride solar cells (Walker *et al.*, 2022 ▸) was studied through a combination of XRF with X-ray-induced current (XBIC)/cross-sectional XBIC measurements. In the case of kesterite [Cu(In,Ga)Se_2_ or Cu_2_ZnSn(S,Se)_4_] solar cells, not only are two multimodal measurements employed (XRF and XBIC) (Huang *et al.*, 2024 ▸; Ossig *et al.*, 2022 ▸) but additional approaches including X-ray beam-induced voltage (Stuckelberger *et al.*, 2017 ▸), X-ray excited optical luminescence (Fevola *et al.*, 2024 ▸), Bragg diffraction (Ulvestad *et al.*, 2019 ▸) and ptychography (Fevola *et al.*, 2024 ▸) have been employed in combination.

These techniques are used to understand perovskite based devices, attracting significant attention in the photovoltaic field. The effect of X-rays during XBIC/XRF measurements (Stuckelberger *et al.*, 2016 ▸; Stuckelberger *et al.*, 2020 ▸) has been investigated, as perovskites are vulnerable to irradiated light sources (electrons, X-rays). Given that the extent of inhomogeneity can be easily influenced by different additive concentrations and external environment conditions, XBIC/XRF measurements provide insights into how the electric properties of devices, incorporating various additive materials such as iron (II) iodide (Poindexter *et al.*, 2018 ▸), alkali halide salts (caesium iodide or rubidium iodide) (Correa-Baena *et al.*, 2019 ▸), as well as under external conditions [*e.g.* continuous light soaking (Li *et al.*, 2020 ▸), and varied humidity (Hidalgo *et al.*, 2023 ▸) and temperature (Rahman *et al.*, 2023 ▸; Tolentino *et al.*, 2023 ▸)], are altered. These endeavours demonstrate that XBIC/XRF is an excellent alternative for observing nano-scale optoelectronic phenomena and chemical alternations within the device configurations.

We have implemented an XBIC setup at the I14 beamline at DLS, enabling corresponding XRF measurements for characterizing photovoltaic devices at the nanoscale. By applying this technique to perovskite solar cells with different additive concentrations, we investigate the correlation between local optoelectronic/chemical features and device performance depending on the additive concentrations. This work provides a multimodal perspective to enable the observation of optoelectronic and chemical properties with high spatial resolution for the field of energy-harvesting devices.

## 2. Experimental details

### 2.1. Samples

The configuration of Cs_0.09_FA_0.91_PbI_3_ perovskite solar cells with phenethyl­ammonium iodide (PEAI) used in this study is as follows: glass/fluorine-doped tin oxide/compact titanium oxide (c-TiO_2_)/mesoporous TiO_2_/PEAI/Cs_0.09_FA_0.91_PbI_3_/PEAI/Spiro-OMeTAD/Au with *n*–*i*–*p* architecture. In terms of the perovskite absorption layer, the different concentrations of methyl­ammonium chloride (MACl) additive (1, 3, 5 and 7%) were added into the perovskite precursor, followed by annealing at 150°C for 10 min.

### 2.2. Measurements

#### 2.2.1. Solar cell characterization

Photovoltaic parameters were evaluated using a Fluxim Litos Lite system, with excitation provided by a Wavelabs Sinus-70 AAA solar simulator under an AM1.5 spectrum. Current–voltage (*J*–*V*) characteristics were recorded in both forward (negative to positive bias) and reverse scans (positive to negative bias) at a rate of 50 mV s^−1^. Device measurements were conducted without any preconditioning. During characterization, the active area of the cells was masked to expose a pixel area of 0.0625 cm^2^. All measurements were carried out in a nitro­gen environment, and no temperature control was applied.

#### 2.2.2. XBIC setup

**Figure 1:**
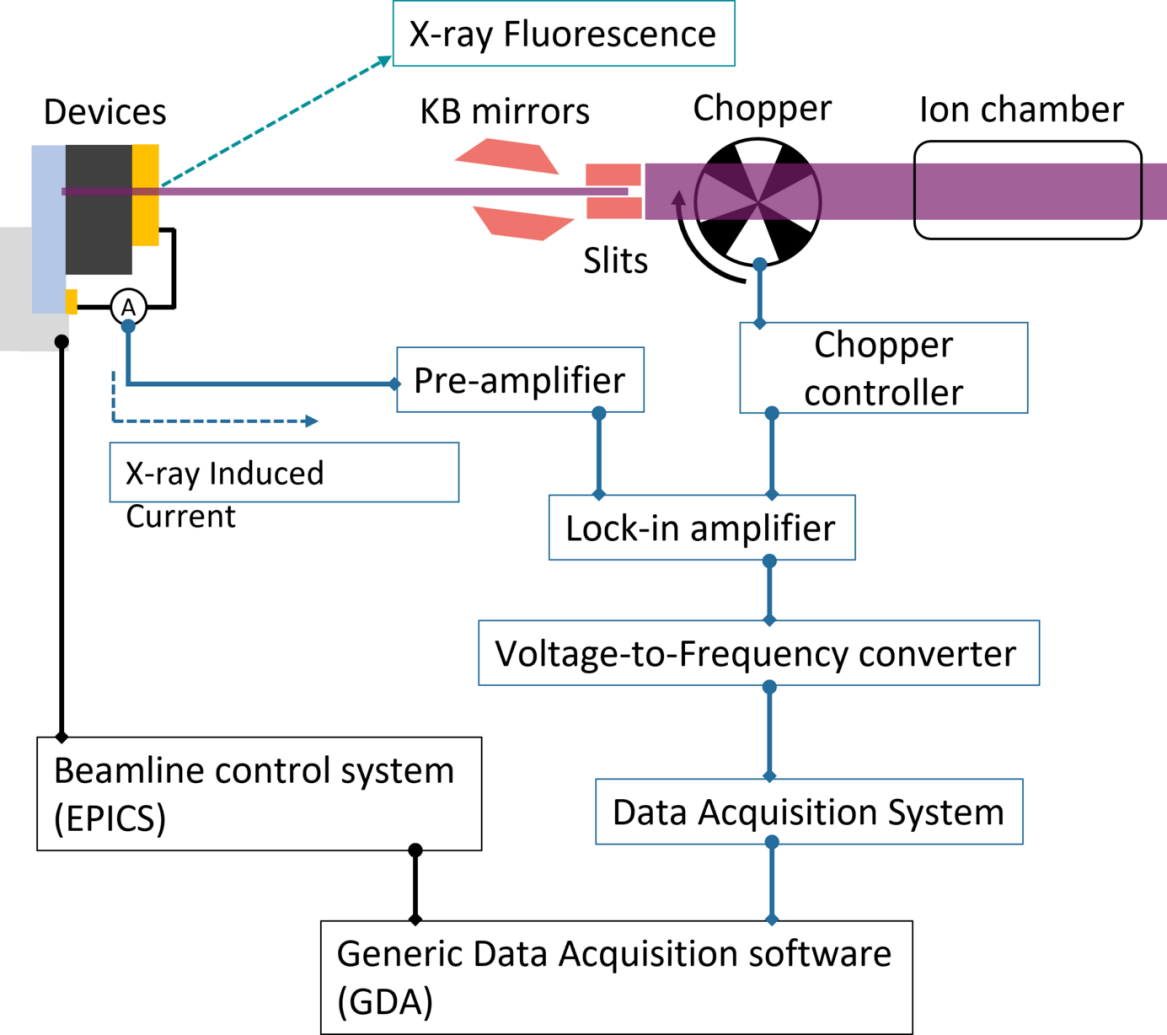
Schematic of XBIC measurements at the I14 beamline.

X-ray nanoprobe measurements were performed at the I14 beamline, DLS (Quinn *et al.*, 2021 ▸), using a focused beam with approximately 50 nm. To measure XBIC while also allowing corresponding XRF measurements, as shown in Fig. 1 ▸, a chopper (MCF10HP, Thorlabs) operating at 738 Hz was installed in the X-ray beam path. The output signal from the chopper controller (Thorlabs) is connected to a lock-in amplifier (MFLI, Zurich Instruments) as the reference signal. Electrically connected devices generate current, which is transferred to the pre-amplifier (SR570, Stanford Research Systems) before passing through the lock-in amplifier to amplify low raw signals. The voltage output from the pre-amplifier is connected to the input of the lock-in amplifier and it is demodulated with the reference signals in the lock-in amplifier. The demodulated voltage output from the lock-in amplifier is transferred to the voltage-to-frequency (V2F) converter (V2F100, Quantum Detectors), followed by a connection to the *Data Acquisition System* (PandA, Quantum Detectors). The data collection is integrated into Diamond’s *Generic Data Acquisition* (*GDA*) software, and we acquire XBIC maps and corresponding XRF maps at the region of interest simultaneously.

#### 2.2.3. XBIC and XRF

XBIC and XRF measurements were performed at 13.5 keV photon energy, to be above the Pb *L*_3_ absorption edge (13.03 keV). XBIC and XRF maps are obtained with a constant velocity scan over a scan size of 10 µm × 10 µm. We first evaluated three acquisition protocols: (i) 100 nm × 100 nm step with 0.015 s dwell [Fig. S1(*a*); avg. 18.6 pA], (ii) 50 nm × 50 nm step with 0.015 s dwell [Fig. S1(*b*); avg. 12.0 pA] and (iii) 100 nm × 100 nm step with 0.030 s dwell [Fig. S1(*c*); avg. 16.8 pA]. Protocol (i) produced the highest average current – and thus the best signal-to-noise at a minimal dose – and was selected for all quantitative XBIC maps, which is consistent with established mitigation strategies (Ossig *et al.*, 2019 ▸; da Silva *et al.*, 2024 ▸). However, an extended 30 µm × 15 µm scan [100 nm × 50 nm, 0.015 s dwell; Fig. S1(*d*)] revealed a measurable current loss in the originally mapped subregion, as confirmed by the line profile in Fig. S1(*e*). Accordingly, all quantitative analyses were restricted to single-pass scans at fresh positions.

For XBIC measurements, the chopper frequency of 738 Hz was set to modulate the X-ray beam. Signals from devices passing through the pre-amplifier with the sensitivity of 20 nA V ^−1^(pre-amplification factor, *A*_PA_) were demodulated in the lock-in amplifier with a low-pass filter frequency of 30.61 Hz (third order) and an amplification scale of 200 (*A*_LIA_). The voltage input range (*R*_v_) and frequency output range (*R*_f_) of the V2F converter are 10 V and 10 MHz, respectively.

**Figure 2:**
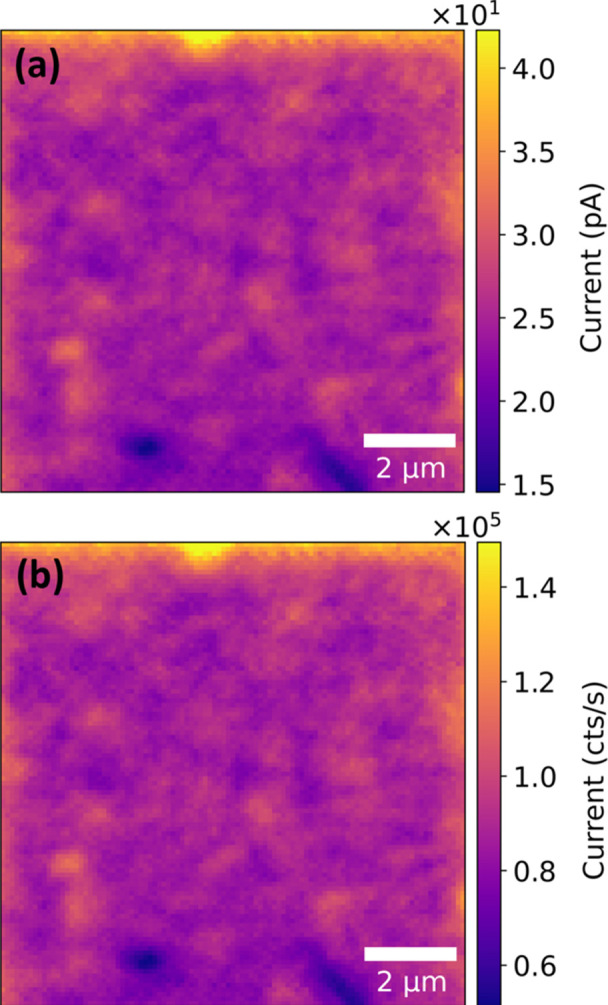
(*a*) Post-processed XBIC signals using conversion equation (1). (*b*) Raw signal from V2F in units of count rates.

Current values, as shown in the map in Fig. 2 ▸(*a*), are calculated from the raw V2F signal (*f*_XBIC_) [see map in Fig. 2 ▸(*b*)] using equation (1) (Ossig *et al.*, 2021 ▸),
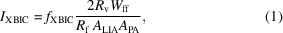
where *W*_ff_ is the constant associated with the modulated waveform. In our case, we used *W*_ff_ = 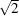 considering the modulated waveform is a sine waveform (Ossig *et al.*, 2021 ▸).

## 3. Results and discussion

**Figure 3:**
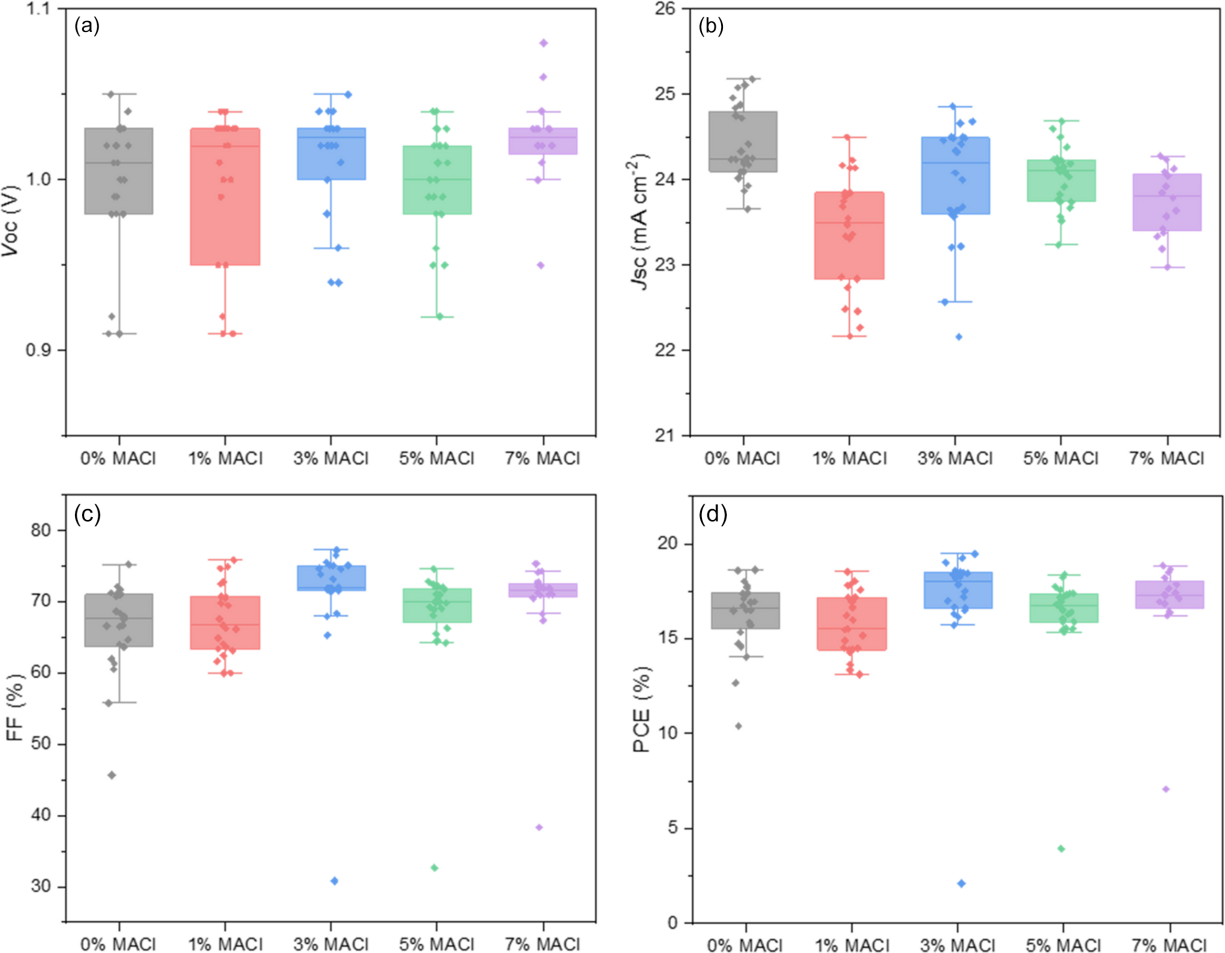
Box plots of device parameters statistics for solar cells (more than 16 devices for each condition) with varying MACl concentrations based on reverse scan data: (*a*) *V*_oc_, (*b*) *J*_sc_, (*c*) FF and (*d*) PCE. *V*_oc_: open-circuit voltage; *J*_sc_: short-circuit current density; FF: fill factor; PCE: power conversion efficiency.

The performance of devices containing different MACl concentrations was investigated using the current–voltage (*J*–*V*) scans, as depicted in Fig. S2. Key photovoltaic parameters were extracted, as summarized in Fig. 3 ▸ and Table 1 ▸, including open-circuit voltage (*V*_oc_), short-circuit current density (*J*_sc_), fill factor (FF) and power conversion efficiency (PCE). While the median *V*_oc_ of the devices with MACl (excluding the 5% MACl) exhibited a modest increase relative to the control sample (0% MACl) at 1.01 V, the overall variation in the median *V*_oc_ remained negligible across the range of MACl concentrations. In contrast, the median *J*_sc_ showed a reduction to 23.5 mA cm^−2^ for 1% MACl condition compared with the control at 24.2 mA cm^−2^. *J*_sc_ recovered at 3% MACl and subsequently declined with further increases in MACl concentration. A similar trend was observed in the median FF, which decreased to 66.7% for 1% MACl condition relative to the control at 67.7%. The median FF significantly improved with higher MACl concentration, peaking at 72.1% for 3% MACl before showing a slight reduction at 5% and 7% MACl. The interplay between *J*_sc_ and FF suggests that a 3% MACl concentration represents an optimal condition, balancing enhanced charge collection (*J*_sc_) and improved charge extraction efficiency (FF) as previously reported by Cao *et al.* (2025 ▸). As a result, the median PCE for 3% MACl concentration increased to 18.0%. These findings highlight the critical role of additive concentration in optimizing photovoltaics. However, the non-linear and ambivalent photovoltaic parameter trends for different MACl conditions underscore the necessity for further characterization to elucidate the underlying effect.

**Figure 4:**
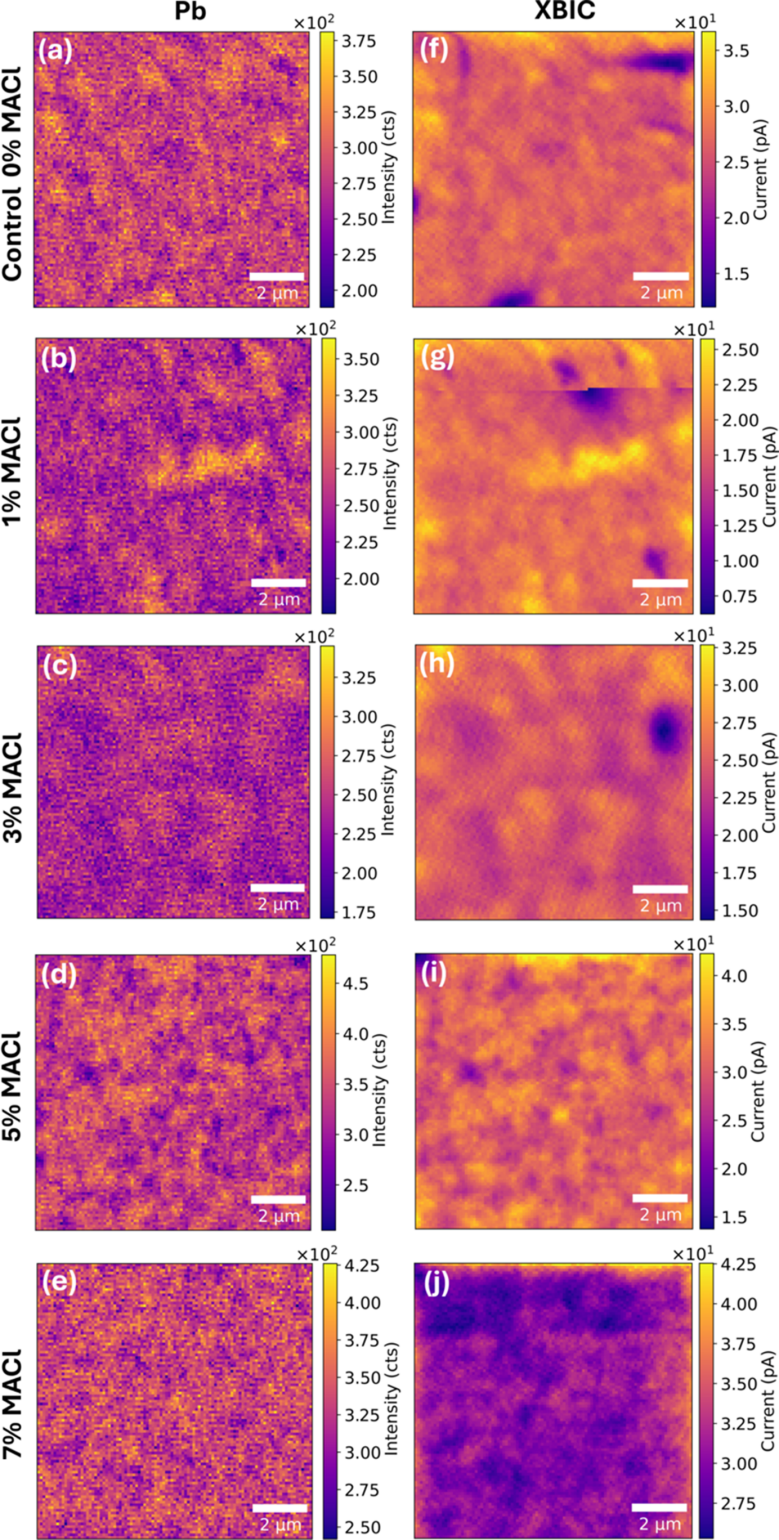
XRF intensity maps of Pb (*a*)–(*e*) and corresponding XBIC maps (*f*)–(*j*) depending on the concentrations of MACl additive: (*a*) and (*f*) without MACl (0% MACl, the control sample), (*b*) and (*g*) with 1% MACl, (*c*) and (*h*) 3% MACl, (*d*) and (*i*) 5% MACl, (*e*) and (*j*) 7% MACl.

To further understand the effect of the MACl additive on the device performance, XBIC/XRF measurements for each device condition are performed. Fig. 4 ▸ presents XBIC maps and corresponding Pb elemental maps (extracted from the XRF) as a function of MACl concentration at 13.5 keV. Note that a few dark regions showing lower current values within the XBIC maps are associated with shunt pathways in the device rather than compositional-dependent results (Kaminski *et al.*, 2004 ▸). Compared with the control sample in Fig. 4 ▸(*a*), after adding 1% MACl, Pb-rich clusters with a large size (approximately 1 µm average size) were observed in Fig. 4 ▸(*b*). Iodine maps can also be extracted from the XRF spectra, hence the iodine signal intensity maps are presented in Fig. S3 along with the map of Pb/I, the ratio of Pb and iodine signal intensities. However, these maps exhibit minimal contrast variations, likely due to the lower sensitivity of XRF at 13.5 keV to iodine signals and the short count times used in this study to reduce beam damage. The observed Pb intensity variations could be influenced by local thickness differences rather than direct compositional changes. Nonetheless, as shown in Fig. 4 ▸(*g*), most Pb-rich clusters exhibited higher current values than their surrounding regions, indicating that Pb spatial distribution remains a key factor affecting charge transport properties. Interestingly, in the case of 5% MACl, rather than observing a few large Pb-clusters, relatively small Pb-clusters comparable to those in the 1% and 3% MACl samples are distributed over the region of interest as presented in Fig. 4 ▸(*d*), causing current variation on smaller areas [Fig. 4 ▸(*i*)]. Although Pb/I maps did not reveal significant compositional contrast (Fig. S3), the increased density of smaller Pb clusters suggests that local Pb spatial variations influence charge collection efficiency, contributing to the observed current distribution. For the highest MACl conditions (7%), the observed Pb-clusters in perovskites with MACl disappeared, becoming similar to compositional variations in the control [Fig. 4 ▸(*d*)]. While the overall current values slightly increased compared with the control [Fig. 4 ▸(*j*)], line profiles as shown in Fig. S4 indicate that this increase was observed both near the edges and within certain internal regions of the sample. This suggests that while beam-induced effects or edge artefacts may contribute to some of the variations, different aspects such as improved charge transport or reduced defect density could also play a role in slightly increased current generation in high MACl conditions.

**Figure 5:**
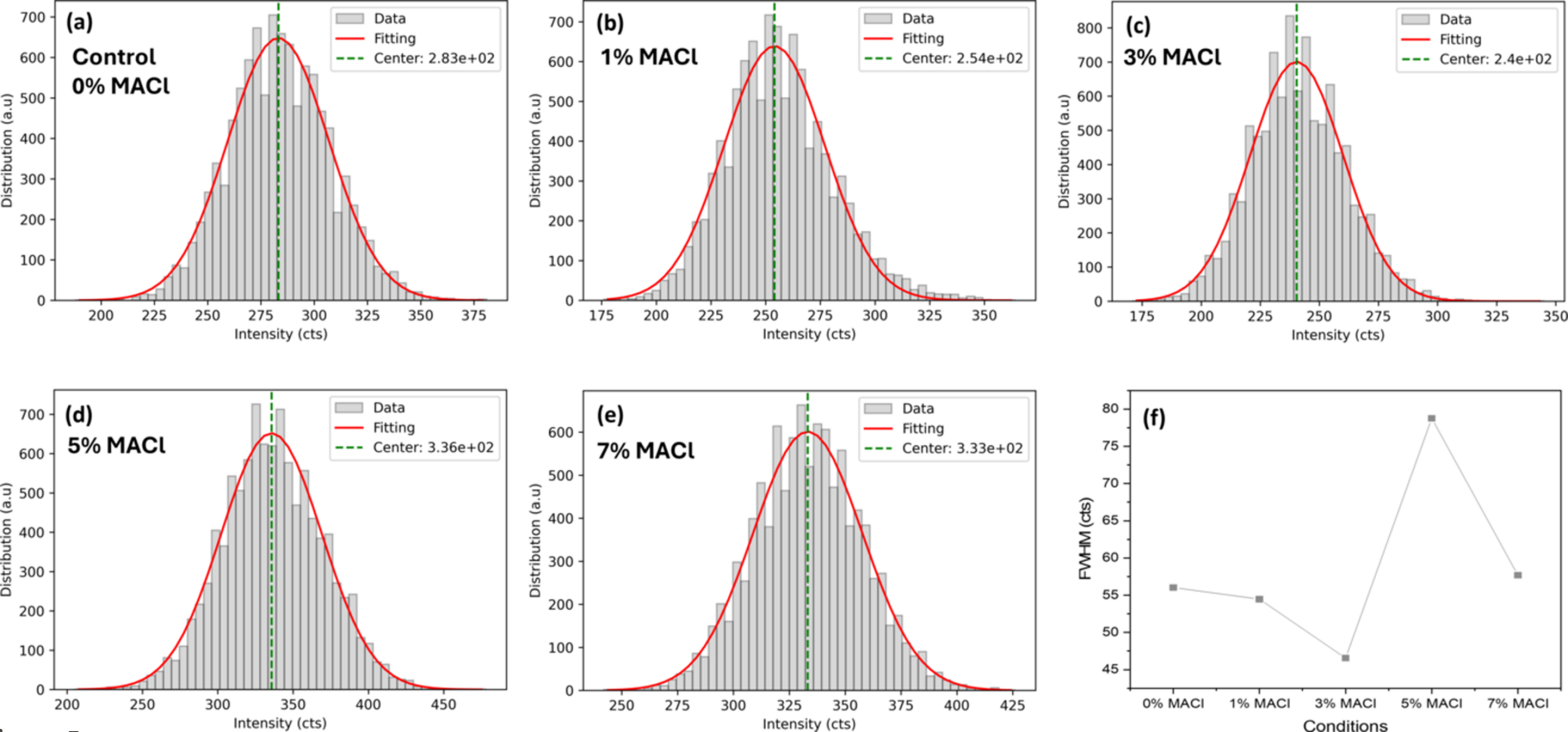
(*a*)–(*e*) Histograms of Pb element distributions from Fig. 4 ▸: (*a*) control sample, (*b*) with 1% MACl, (*c*) 3% MACl, (*d*) 5% MACl and (*e*) 7% MACl. (*f*) FWHM values corresponding to the MACl concentrations in (*a*)–(*e*), derived through Gaussian fitting. Additional values related to Gaussian fitting are provided in Table 2 ▸.

The histograms obtained from Pb element maps as shown in Figs. 5 ▸(*a*)–5 ▸(*e*) were analysed quantitatively under each condition through Gaussian distribution fitting. The parameters obtained from the fitting for each condition are summarized in Table 2 ▸, and the FWHM of the histogram was determined based on the standard deviation (σ) values [as presented in Fig. 5 ▸(*f*)] following a well established relationship described by Tavernier (2009 ▸). The FWHM value reached its minimum at 3% MACl concentration, indicating a more homogeneous Pb distribution, while the FWHM almost doubled at 5% MACl, indicating a much more heterogeneous Pb distribution.

**Figure 6:**
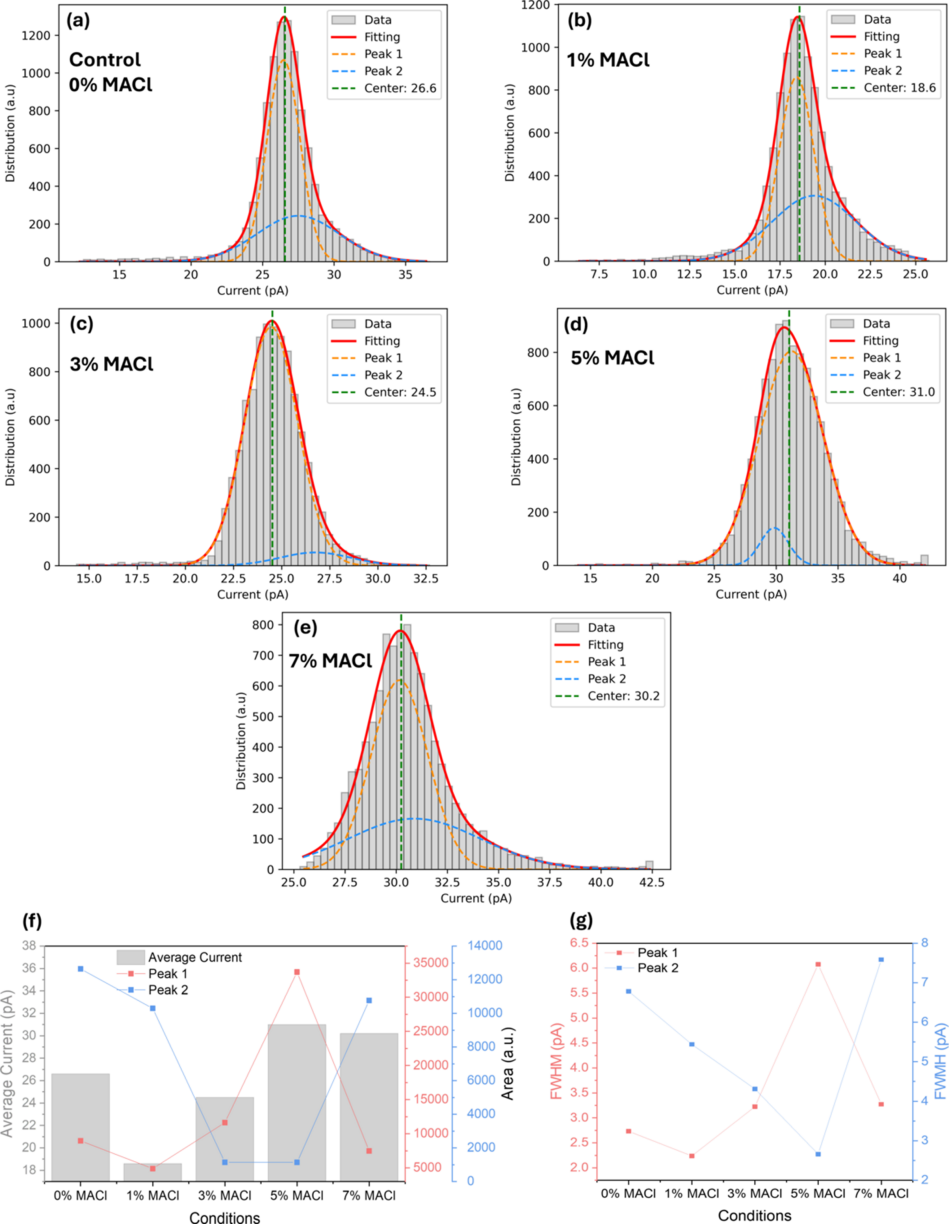
(*a*)–(*e*) Histograms of generated currents from Fig. 4 ▸: (*a*) control sample, (*b*) with 1% MACl, (*c*) 3% MACl, (*d*) 5% MACl and (*e*) 7% MACl. After the Gaussian deconvolution of peaks presented by orange (Peak 1, which is located closer to the average current values) and sky-blue lines (Peak 2, which is positioned further away from the average current values compared with Peak 1), a fitted curve (red line) could be obtained. The green line in each panel indicates average currents from the acquired XBIC maps. (*f*) Average current and occupied peak area, and (*g*) FWHM values regarding the concentration of additives acquired from (*a*)–(*e*) of Fig. 6 ▸ with Gaussian fitting. Additional values related to Gaussian fitting are placed in Table 3 ▸.

Through a similar approach for XBIC maps, the average generated current values from measured regions were also acquired as shown in Fig. 6 ▸. The average current of the control sample is 26.6 pA. However, the average current value significantly dropped with the addition of 1% MACl. The generated current value seems to be recovered with the addition of 3% MACl (24.5 pA) while a higher generated current value was observed at 5% MACl (31 pA). At higher MACl concentration (7%), the average current slightly decreased to 30.2 pA. Compared with Pb element maps, the single Gaussian fitting failed to adequately capture the tail regions of some of the measured data, necessitating the introduction of a multi-peak Gaussian fitting approach as shown in Figs. 6 ▸(*a*)–6 ▸(*e*). The parameters obtained by utilizing this fitting are shown in Table 3 ▸. For each sample, two peaks were identified: Peak 1, which is closer to the average current values; and Peak 2, which has a larger distance from the average current values than Peak 1.

Figs. 6 ▸(*f*) and 6 ▸(*g*) present the tendency of the area and FWHM of individual peaks as a function of MACl concentrations. The area and FWHM of Peak 1, which predominantly appears in the current maps, exhibit a trend similar to that of the FWHM of Pb elements, further suggesting a strong relationship between Pb spatial uniformity and current distribution. At 3% MACl, the narrower FWHM of Peak 1 reflects uniform current generation, consistent with enhanced charge collection efficiency and the highest FF. Conversely, at 5% MACl, the broader FWHM of Peak 1 indicates increased heterogeneity, correlating with reduced FF despite the higher average current. When examining Peak 2, which accounts for the minority in the histogram, a decrease in both its occupied area and FWHM can be observed at 5% MACl concentration. However, this reduction in the area and FWHM of Peak 2 at 5% MACl does not appear to fully mitigate the effects of the increased heterogeneity observed in Peak 1 and Pb maps, as reflected in the device performance trends.

The origins of these peaks cannot be determined solely based on the current distribution data. However, previous studies on current (Thomas *et al.*, 2020 ▸) and surface potential (Ding *et al.*, 2019 ▸) distributions in various materials have linked the presence of two distinct peaks in histograms to structural differences. For instance, in self-assembled monolayers, such peaks were associated with structural variations, while in MoS_2_/PbI_2_ heterostructures, they were attributed to variations in work function and compositional differences. In the context of mixed halide perovskites, the reduction in the occupied area and FWHM of Peak 2 at 5% MACl concentration suggests reduced secondary phases and improved local structural uniformity. However, the broader FWHM of Peak 1 and Pb maps indicates that the global structural heterogeneity may still limit device performance, particularly in terms of FF and PCE. Studies, such as that by Wang *et al.* (2024 ▸) have shown that MACl improves crystallization kinetics, leading to larger grain sizes, better crystallinity and reduced defect densities. This suggests that optimal MACl concentrations can suppress secondary phases or structurally disordered regions. At 3% MACl, these effects appear to be maximized, yielding the most uniform Pb distribution, enhanced charge transport and superior device performance. In contrast, higher MACl concentrations, such as 5%, introduce structural stress or heterogeneity that compromises overall device efficiency despite locally enhanced current generation.

Based on these observations, Peak 1, which dominates the central distribution, likely represents regions with uniform crystallinity and fewer defects, while Peak 2, showing greater deviations, may correspond to areas with compositional heterogeneity or secondary phases. The reduction in FWHM and area of Peak 2 at 5% MACl concentration supports the hypothesis that MACl minimizes structural inconsistencies; however, the accompanying increase in heterogeneity reflected in Peak 1 and Pb maps highlights the challenges of balancing local and global uniformity for optimal device performance.

**Figure 7:**
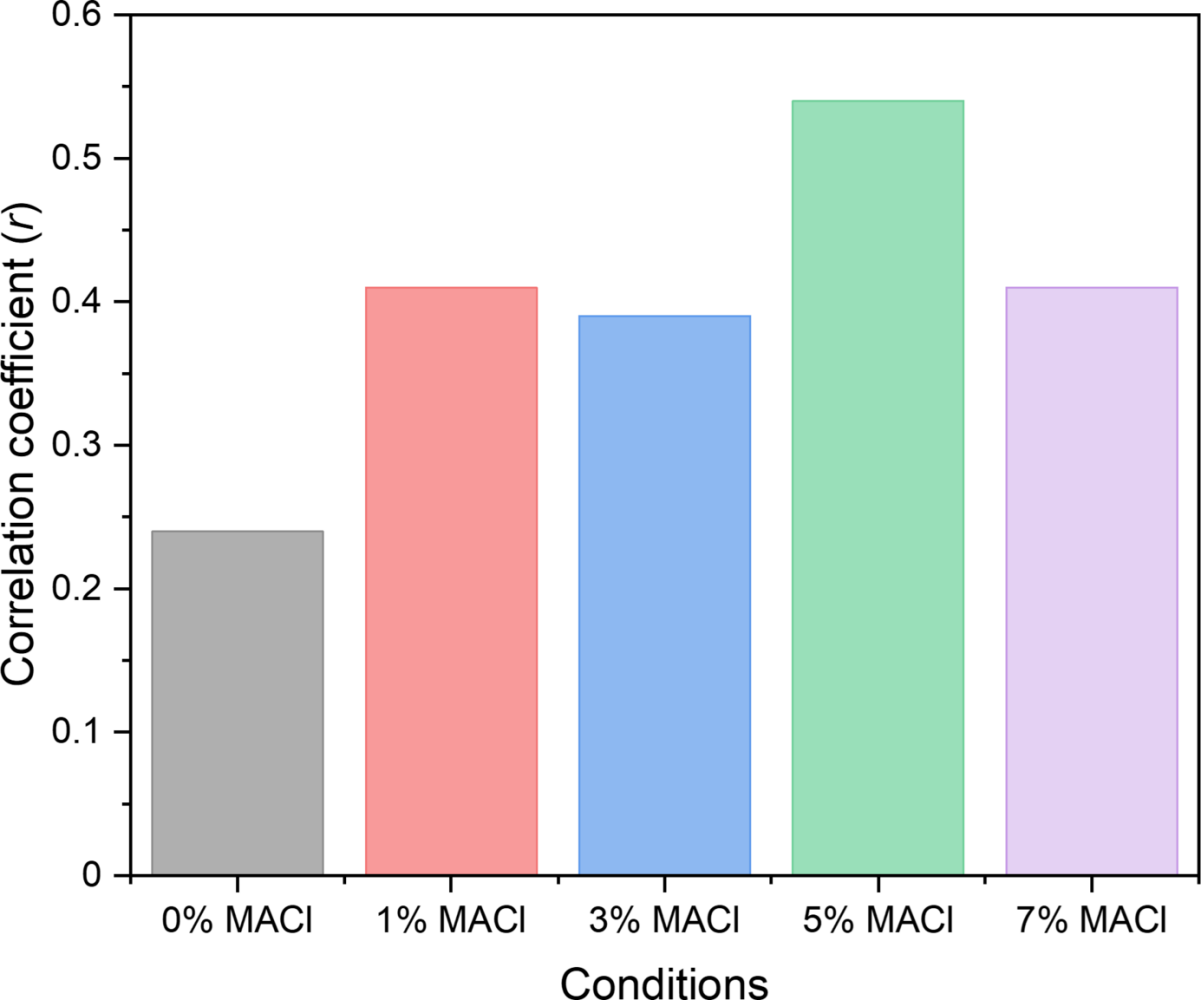
Pearson product–moment correlation coefficients (*r*) from 2d-KDE plots from Fig. S5. Additional information related to this correlation coefficient is described in the supplementary note following Fig. S5.

To investigate the correlations of nanoscale optoelectronic performance with Pb XRF signal intensity variations across the entire map, two-dimensional kernel density estimation (2 d-KDE) analysis was performed (see Fig. S5), and the Pearson product–moment correlation coefficients (*r*) as a function of MACl concentrations were obtained as shown in Fig. 7 ▸. Regardless of different MACl concentrations, all conditions show positive correlation coefficients between the current and the Pb signal intensity. Given that *r* > 0.7 describes a strong positive correlation (Schober *et al.*, 2018 ▸), the generated current values demonstrate a moderate dependence on Pb element, reinforcing the role of Pb spatial uniformity in enhancing device performance.

At 5% MACl, the correlation coefficient reached its maximum (*r* = 0.54), suggesting that increased Pb-related structural improvements contributed to enhanced local current generation, as observed in the XBIC results (average current of 31 pA). However, the corresponding device performance was suboptimal due to broader FWHM of Pb and FWHM of XBIC maps, respectively, which likely led to uneven charge transport and recombination losses. In contrast, the 3% MACl condition, which showed a slightly lower correlation coefficient (*r* = 0.39), yielded the best device performance in terms of PCE, FF and *J*_sc_. This indicates that, while a high correlation between Pb content and current generation is beneficial, the global uniformity of Pb distribution, as reflected in the narrower Pb FWHM, plays a more critical role in enhancing overall device efficiency.

At 7% MACl, the correlation coefficient dropped back to *r* = 0.41. There can be several causes for this correlation reduction in high MACl conditions; one is the gradient growth of photo-inactive materials (effect on resultant *J*_sc_ and FF) such as non-perovskite structures (*e.g.* 2H perovskites or 4H hexagonal polytype phases) observed in the literature (Chang *et al.*, 2023 ▸; Shen *et al.*, 2023 ▸; Wang *et al.*, 2024 ▸) through XRD. It is likely that a combination of effects, such as an increase in the efficiency of charge transport (Kim *et al.*, 2019 ▸) or a decrease in defects in Pb due to an increase in grain size, along with a decrease in the targeted crystalline α-phase give rise to the generated current as measured in Fig. 6 ▸(*f*) for high MACl conditions.

## 4. Conclusions

In this work, to demonstrate the benefits of correlating hard X-ray nanoprobe measurement techniques and corresponding optoelectronic properties of actual devices at the nanoscale, we expanded our measurement capabilities to enable XBIC measurements at the I14 beamline, DLS. Using the newly implemented technique (multi-modal XRF/XBIC measurements) we analysed perovskite solar cell devices to examine the correlation between local elemental concentration and the generated current as a function of the MACl additive concentrations. These results demonstrate the *operando* capabilities of the hard X-ray nanoprobe beamline at DLS for probing the micro to nanoscopic compositional–optoelectronic properties of devices composed of energy-harvesting materials, and potential application to investigate the performance and stability of devices in the future.

## 5. Related literature

The following references, not cited in the main body of the paper, have been cited in the supporting information: Di Leo & Sardanelli (2020 ▸); Puth *et al.* (2014 ▸).

## Supplementary Material

Supporting information. DOI: 10.1107/S1600577525006034/ing5013sup1.pdf

## Figures and Tables

**Table 1:** Photovoltaic parameters with different concentrations of MACl (from Fig. 3 ▸), extracted from the reverse *J–V* scan The statistical values presented are median values. The median absolute deviations are reported in parentheses.

Conditions	No. of devices	*V*_oc_ (V)	*J*_sc_ (mA cm^−2^)	FF (%)	PCE (%)
0% MACl	24	1.01 (±0.02)	24.2 (±0.2)	67.7 (±3.5)	16.6 (±0.9)
With 1% MACl	23	1.02 (±0.02)	23.5 (±0.6)	66.7 (±3.8)	15.5 (±1.2)
With 3% MACl	22	1.03 (±0.01)	24.2 (±0.5)	72.1 (±2.6)	18.0 (±0.9)
With 5% MACl	24	1.00 (±0.02)	24.1 (±0.2)	69.9 (±1.9)	16.7 (±0.7)
With 7% MACl	16	1.03 (±0.01)	23.8 (±0.3)	71.5 (±0.8)	17.3 (±0.7)

**Table 2:** Parameters of Gaussian distribution fitting for Pb maps μ indicates the mean of the distribution, σ represents the standard deviation.

	μ	Area	Height	σ	*R* ^2^
0% MACl	283.19	38663.07	648.24	23.79	0.9729
With 1% MACl	254.01	37043.89	638.96	23.13	0.9628
With 3% MACl	240.36	34727.63	700.95	19.77	0.9628
With 5% MACl	335.68	54674.06	652.19	33.44	0.9801
With 7% MACl	333.24	36901.85	601.08	24.49	0.9691

**Table 3:** Parameters of Gaussian distribution fitting for XBIC maps μ indicates the mean of the distribution, σ represents the standard deviation.

	Index	μ	Area	Height	σ	*R* ^2^
0% MACl	Peak 1	26.47	8965.47	3094.74	1.16	0.9988
	Peak 2	27.49	12641.51	1752.37	2.88	
With 1% MACl	Peak 1	18.42	4896.51	2052.61	0.95	0.9979
	Peak 2	19.39	10295.93	1774.53	2.31	
With 3% MACl	Peak 1	24.46	11628.33	3383.18	1.37	0.9971
	Peak 2	26.75	1144.23	249.28	1.83	
With 5% MACl	Peak 1	31.24	33702.38	5206.43	2.58	0.9976
	Peak 2	29.85	1136.65	400	1.13	
With 7% MACl	Peak 1	30.16	7465.95	2148.77	1.39	0.9924
	Peak 2	30.89	10772.41	1336.55	3.22	

## Data Availability

The data underpinning the results presented in this paper will not be publicly available at this time. However, on publication, the data processing code used for the analysis in this paper will be made available on a Zenodo or GitHub repository.
