# Supplementary material for: Multimodal hard X-ray nanoprobe techniques for operando investigations of photovoltaic devices
Source: J Synchrotron Radiat. 2025 Aug 19;32(Pt 5):1211–9. doi: 10.1107/S1600577525006034 (PMC12416418; doi:10.1107/S1600577525006034)
Supplement: Supplementary file 1 [file s-32-01211-sup1.pdf]

JOURNAL OF SYNCHROTRON RADIATION

Volume 32 (2025)

Supporting information for article:

Multimodal hard X-ray nanoprobe techniques for *operando* investigations of photovoltaic devices

Eunyoung Choi, Sarah Wieghold, Carlo A. R. Perini, Yanqi Luo, Sanggyun Kim, Juan-Pablo Correa-Baena, Samuel D. Stranks and Julia E. Parker

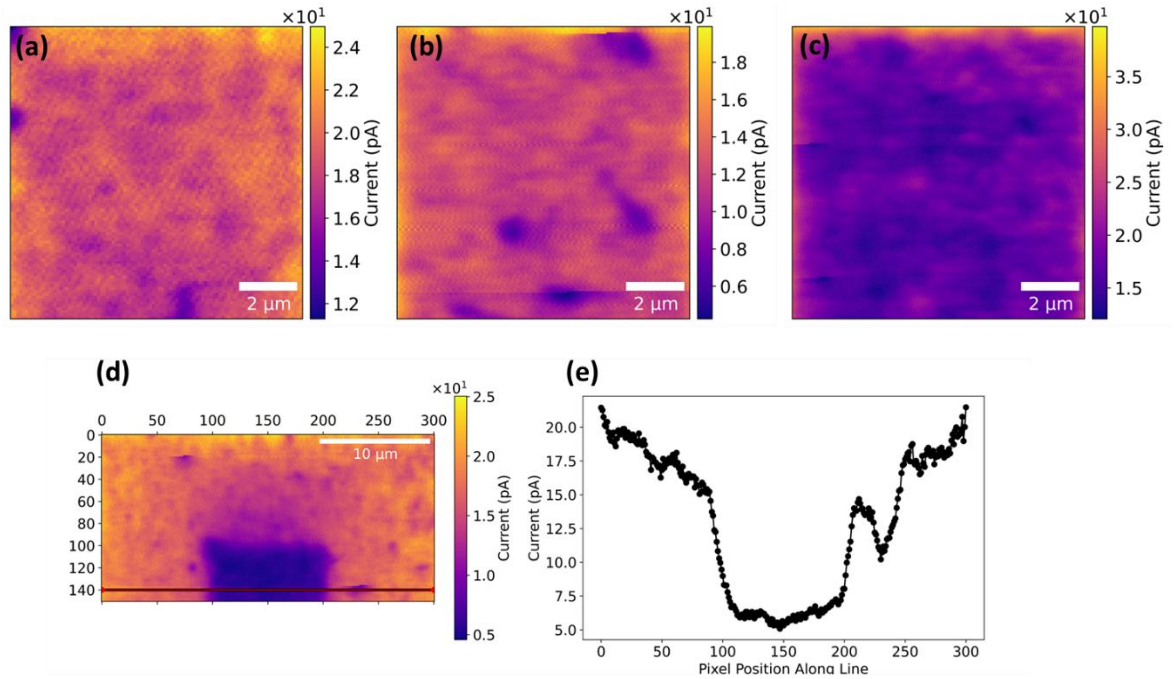

Figure S1 XBIC maps of 5% MACl–treated samples acquired under varied scan conditions: (a) 10 μm × 10 μm area, 100 nm × 100 nm step size, 0.015 s dwell time; (b) 10 μm × 10 μm area, 50 nm × 50 nm step size, 0.015 s dwell time; (c) 10 μm × 10 μm area, 100 nm × 100 nm step size, 0.030 s dwell time. (d) extended 30 μm × 15 μm scan (100 nm × 50 nm, 0.015 s dwell), illustrating overlap of the original 10 μm × 10 μm region. (e) line profile extracted from the overlap region in (d), quantifying the reduction in XBIC signal due to prolonged exposure.

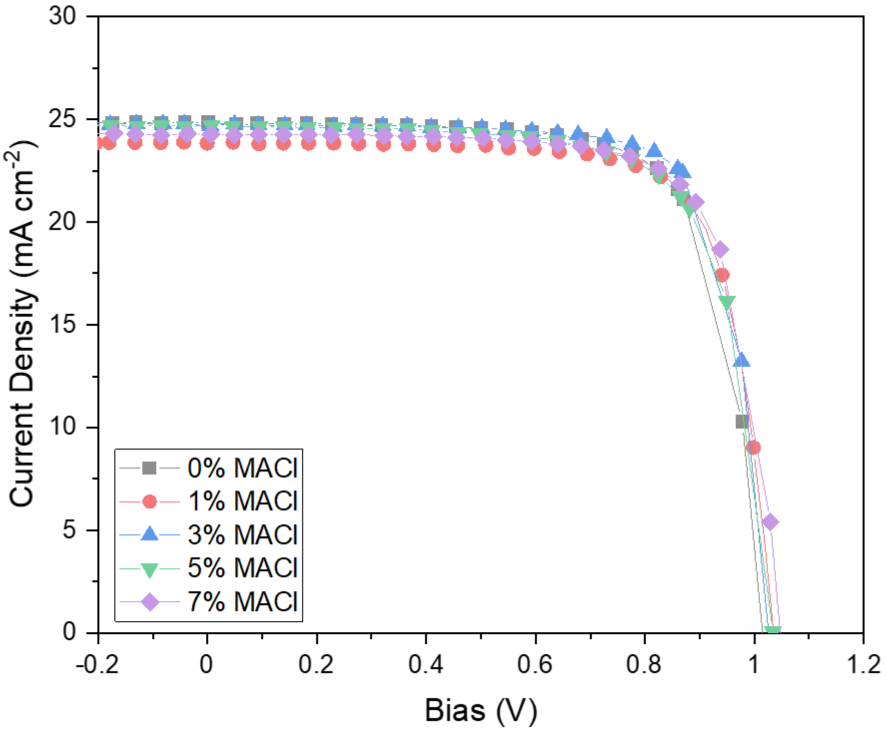

Figure S2 *J-V* curve of the champion device for different MACl concentrations.

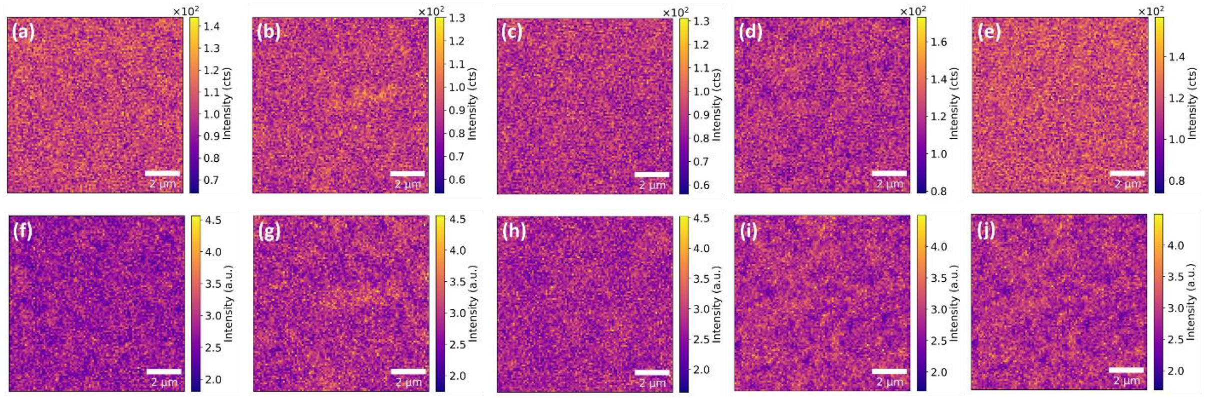

Figure S3 (a-e) XRF intensity maps of I at different MACl concentrations (0%, 1%, 3%, 5%, 7%), (f-j): Pb/I ratio maps at different MACl concentrations (0%, 1%, 3%, 5%, 7%).

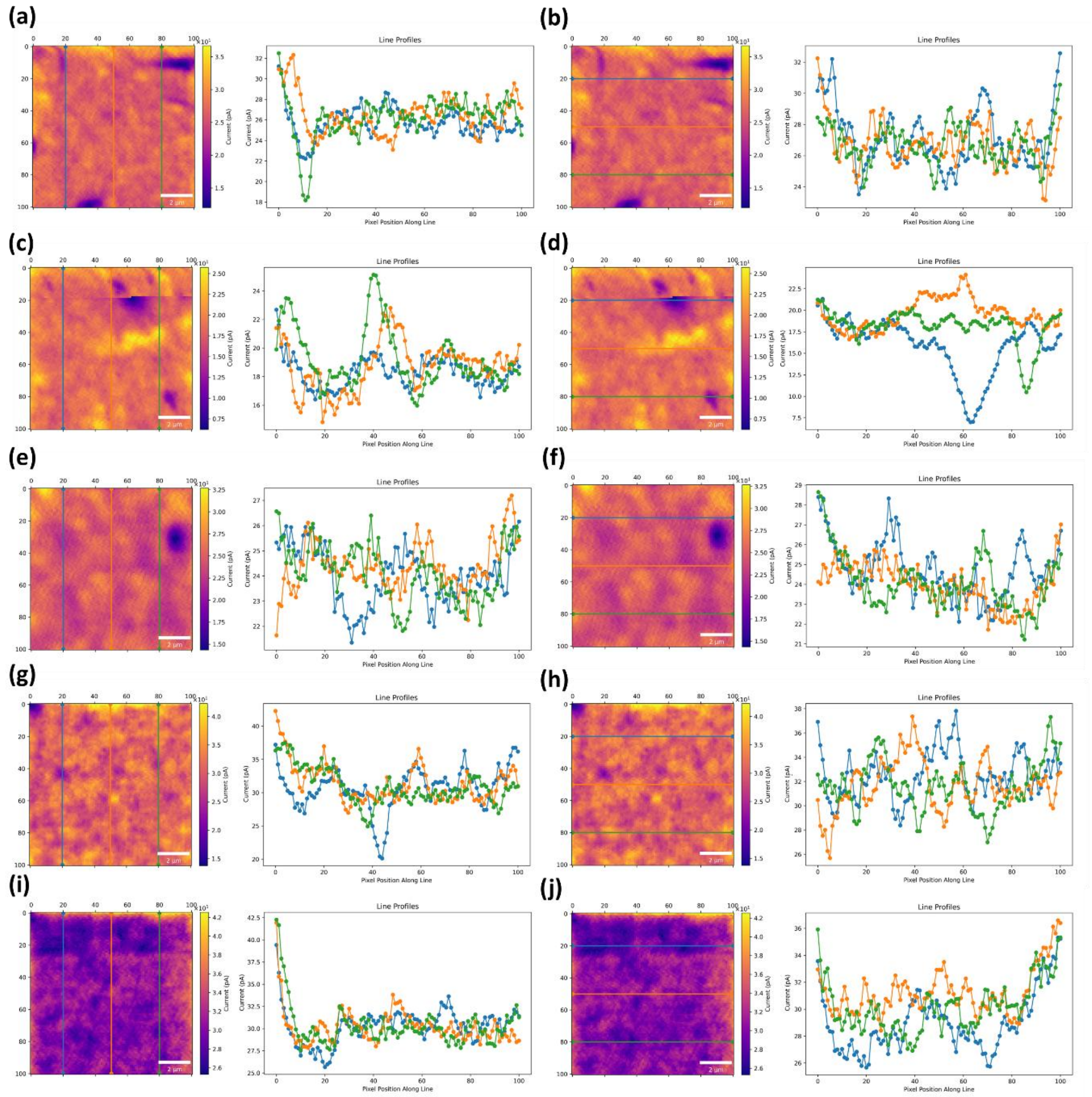

Figure S4 Line profiles from XBIC maps across different MACl concentrations. The left column (a, c, e, g, i) shows images with three vertical lines indicating the positions where vertical line profiles were extracted, along with their corresponding XBIC variations. The right column (b, d, f, h, j) presents similar analyses for horizontal line profiles, where three horizontal lines mark the extraction positions. Each row represents different MACl concentrations in increasing order: 0% (a, b), 1% (c, d), 3% (e, f), 5% (g, h), and 7% (i, j).

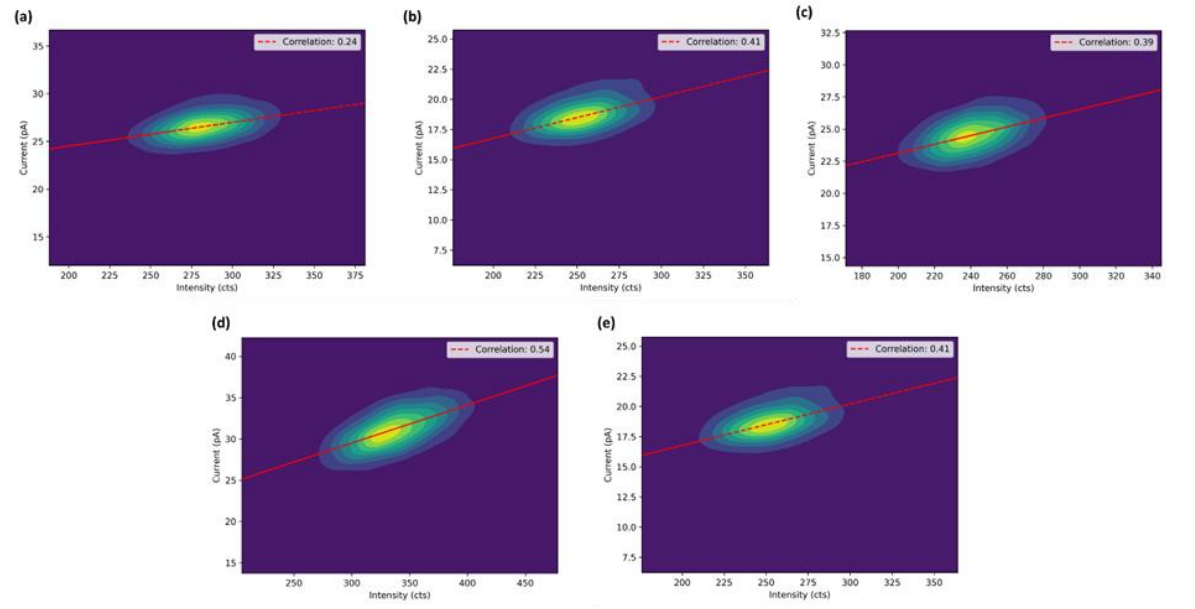

Figure S5 Line profiles from XBIC maps across different MACl concentrations. The left column (a, c, e, g, i) shows images with three vertical lines indicating the positions where vertical line profiles were extracted, along with their corresponding XBIC variations. The right column (b, d, f, h, j) presents similar analyses for horizontal line profiles, where three horizontal lines mark the extraction positions. Each row represents different MACl concentrations in increasing order: 0% (a, b), 1% (c, d), 3% (e, f), 5% (g, h), and 7% (i, j).

S1. Supplementary note for Figure S5 and Figure 7

We used the Pearson product-moment correlation formula which is one of the most used correlation approaches to acquire the Pearson product-moment correlation coefficients (*r*) below the equation:

$$r = \frac{\sum(x-\bar{x})(y-\bar{y})}{\sqrt{(x-\bar{x})^2\,(y-\bar{y})^2}} \qquad \text{(S1)}$$

where r is the Pearson correlation coefficient, x and y are x variable and y variable values, respectively. $\bar{x}$ is the mean of x variable. $\bar{y}$ is the mean of y variable (Puth *et al.*, 2014).
(In this study, x variable is Pb elements variation, while y variable is generated current values).

Given that the *r* value acquired from the above equation indicates a linear relationship between two variables (x and y), the *r* value ranges exist between -1 and 1. If r indicates 0, there is no linear relationship between the variables. There is a positive linear correlation when $0 < r < 1$, whereas there is a negative linear correlation when $-1 < r < 0$. The closer the absolute value of *r* ($|r|$) is to 1, the stronger correlation (Schober *et al.*, 2018).
The *r* values were obtained through the *corrcoef function* (the Pearson product-moment correlation coefficient return function) in the module of *numpy*, and the linear regression function (*linregress*) in the *scipy.stats* module also obtained the *p* value as well as the *r* value obtained earlier.

To estimate the statistical significance of the correlation, the *p*-value is considered before using *r* value. Because of the double precision limit, we obtained values of 1% MACl, 3% MACl, and 5% MACl with *p* values of 0 and $p < 10^{-133}$ for the content sample and $p < 10^{-67}$ for 7% MACl. Since the *p* value for all samples is much smaller than the threshold ($p < 0.05$) (Di Leo & Sardanelli, 2020) which is considered statistically significant, we can use correlations of the calculated Pearson product-moment correlation coefficients.

References for supporting information

Di Leo, G. & Sardanelli, F. (2020). *European Radiology Experimental* **4**, 18.
Puth, M.-T., Neuhäuser, M. & Ruxton, G. D. (2014). *Animal Behaviour* **93**, 183-189.
Schober, P., Boer, C. & Schwarte, L. A. (2018). *Anesthesia & Analgesia* **126**.
